# Evaluation of SARS-CoV-2 Vaccine-Induced Antibody Responses in Patients with Neuroimmunological Disorders: A Real-World Experience

**DOI:** 10.3390/diagnostics14050502

**Published:** 2024-02-26

**Authors:** Hyunjin Ju, Jin Myoung Seok, Yeon Hak Chung, Mi Young Jeon, Hye Lim Lee, Soonwook Kwon, Sunyoung Kim, Ju-Hong Min, Byoung Joon Kim

**Affiliations:** 1 Neuroscience Center, Samsung Medical Center, Seoul 06351, Republic of Korea; joohj365@gmail.com (H.J.);; 2 Department of Neurology, Samsung Medical Center, Sungkyunkwan University School of Medicine, Seoul 06351, Republic of Korea; 3 Department of Neurology, Soonchunhyang University Seoul Hospital, Soonchunhyang University College of Medicine, Seoul 04401, Republic of Korea; 4 Department of Neurology, Soonchunhyang University Hospital Cheonan, Soonchunhyang University College of Medicine, Cheonan 31193, Republic of Korea; 5 Department of Neurology, Korea University Guro Hospital, Korea University College of Medicine, Seoul 08308, Republic of Korea; 6 Department of Neurology, Inha University Hospital, Inha University College of Medicine, Incheon 22332, Republic of Korea; 7 Department of Neurology, Ulsan University Hospital, University of Ulsan College of Medicine, Ulsan 44033, Republic of Korea; mdneurosk@gmail.com; 8 Department of Health Sciences and Technology, Samsung Advanced Institute for Health Sciences & Technology (SAIHST), Sungkyunkwan University, Seoul 06351, Republic of Korea

**Keywords:** COVID-19 vaccines, autoimmune diseases of the nervous system, COVID-19, immunomodulating agents, immunosuppressive agents

## Abstract

This study evaluates the antibody responses to SARS-CoV-2 vaccines in patients with neuroimmunological disorders (pwNID) who are receiving immunomodulating treatments, compared to healthy individuals. It included 25 pwNID with conditions such as optic neuritis, neuromyelitis optica spectrum disorder, multiple sclerosis, myasthenia gravis, and polymyositis, as well as 56 healthy controls. All participants had completed their full SARS-CoV-2 vaccination schedule, and their blood samples were collected within six months of their last dose. The concentration of anti-SARS-CoV-2 IgG antibodies was measured using an enzyme-linked immunosorbent assay. The results showed that pwNID had significantly lower antibody titers (58.4 ± 49.2 RU/mL) compared to healthy individuals (81.7 ± 47.3 RU/mL). This disparity persisted even after adjusting for age and the interval between the final vaccination and sample collection. A notable correlation was found between the use of immunomodulating treatments and reduced antibody levels, whereas mRNA vaccines were linked to higher antibody concentrations. The conclusion of this study is that immunomodulating treatments may reduce the effectiveness of SARS-CoV-2 vaccines in pwNID. This insight is crucial for healthcare providers in designing vaccination strategies and managing treatment plans for pwNID on immunomodulating therapies, highlighting the need for personalized approaches in this subgroup.

## 1. Introduction

Widespread vaccination efforts against coronavirus disease 2019 (COVID-19) have been implemented to build community immunity and mitigate serious outcomes from the diseases [1,2]. In South Korea, the BNT162b2 (Pfizer-BioNTech) and ChAdOx1-S/nCoV-19 (AstraZeneca) vaccines were initially introduced, followed by the utilization of two other vaccines in 2022, including Ad26.COV2.S (Janssen) and mRNA-1273 (Moderna) vaccines [3]. As the COVID-19 pandemic evolved with new variants of SARS-CoV-2 coronavirus, public perception towards the virus also shifted. Despite these changes, the Omicron variant still presented a notable case fatality rate, indicating the persistent risk and impact of the virus [4].

Neuroimmunological disorders are characterized by their inflammatory or immune-mediated pathomechanisms and are commonly managed with treatments that involve immunomodulatory or immunosuppressive medications [5]. Due to these characteristics, COVID-19 and neuroimmunological disorders mutually influence each other. COVID-19 infection is known to be related to neuroimmunological mechanisms [6]. Reports have indicated that COVID-19 can lead to various neurological complications, potentially caused by direct invasion of the virus or the indirect consequences of the postinfectious immune response [6,7]. Furthermore, COVID-19 infection can cause neuroimmunological disorders. There have been reports that Guillain–Barre syndrome, multiple sclerosis (MS), neuromyelitis optica spectrum disorder (NMOSD), myelin oligodendrocyte glycoprotein antibody-associated disease (MOGAD), or unusual central nervous system (CNS) demyelinating events can occur post-COVID-19, although it remains uncertain whether these are causal links or coincidental findings [8,9,10].

COVID-19 infection could be severe in patients with neuroimmunological disorders. A Danish cohort study indicated an increased risk of COVID-19-related hospitalization and mortality in these patients [11]. The patients with MS receiving anti-CD20 therapies show severe breakthrough COVID-19 infection [12,13]. Myasthenia gravis (MG) patients also exhibit worse outcomes from COVID-19 infection [14]. Although the exact mechanisms are not fully understood, it is believed that the characteristics of neuroimmunological disorders and the use of immunosuppressive drugs are influential factors. Immune-mediated neuromuscular disorders can lead to respiratory complications due to disease activity. Consequently, the risk of mechanical ventilation and mortality can be exacerbated by COVID-19 infection [15].

Additionally, COVID-19 infection can have an impact on neuroimmunological disorders. The occurrence and severity of COVID-19 infection are associated with worsening clinical disability in MS patients [16] and are also related to the exacerbation or unmasking of underlying autoimmune neuromuscular disorders [15]. Reports have indicated that relapses of NMOSD and MOGAD occur after COVID-19 infection, although the risk of relapse due to treatment interruption may be higher [17,18]. However, recent reports regarding a broader perspective have suggested that COVID-19 has no impact on disease activity or disease course in patients with MS [19,20]. Further long-term studies are needed to gain a comprehensive understanding of the clinical impact of COVID-19 infection on neuroimmunological disorders.

Since these vaccines are non-live vaccines, they could potentially be administered safely to patients with neuroimmunological disorders receiving immunosuppressive therapies. The World Health Organization (WHO) advocates for immunocompromised individuals to receive the severe acute respiratory syndrome coronavirus 2 (SARS-CoV-2) vaccine and its booster shot [21,22]. However, the efficacy of SARS-CoV-2 vaccines in immunocompromised individuals remains a crucial concern. Neuroimmunological disorders might be associated with immune dysfunction related to the underlying disorders or use of immunomodulating/immunosuppressive therapies. Previous studies have demonstrated blunted serologic responses in patients who are treated with immunosuppressants, especially B-cell depleting agents, fingolimod, or mycophenolate mofetil [23,24,25,26]. Patients treated with anti-TNF drugs may exhibit a rapid decrease in anti-SARS-CoV-2 antibodies, potentially leading to undetectable levels within three months after receiving the second mRNA vaccine dose [27]. Metanalyses have confirmed that patients with immune-mediated inflammatory diseases tend to exhibit lower rates of seroconversion after COVID-19 vaccination, with certain classes of drugs linked to decreased seroconversion effectiveness [28].

The interactions between COVID-19, its vaccination, and neuroimmunological disorders are complex and multifaceted, requiring thorough investigation and thoughtful clinical consideration. However, there is a paucity of studies on Korean patients with neuroimmunological disorders who are undergoing treatment with immunomodulating therapies.

In the present study, we aimed to assess the antibody responses generated by SARS-CoV-2 vaccines in patients with neuroimmunological disorders based on their clinical status and treatment.

## 2. Materials and Methods

### 2.1. Study Population and Data Collection

From October 2021 to February 2022, we prospectively enrolled patients with neuroimmunological disorders receiving immunomodulating treatment in Samsung Medical Center. Healthy volunteers were also enrolled for comparison. All enrolled subjects had received the standard dose of COVID-19 vaccination, as initially recommended in Korea, with a single dose of Ad26.COV2.S and two doses of BNT162b2, ChAdOx1-S/nCoV-19, and mRNA-1273, with or without additional booster shots. Participants who had previously contracted COVID-19 infection, had hematologic malignancies potentially affecting the immune system, or had undergone any form of treatment that alters immune system function, such as cancer treatment, were excluded.

Blood samples from the enrolled subjects were collected within six months following their most recent vaccinations. We collected the clinical characteristics of the patients, including their demographic data, type of neuroimmunological disorders, immunomodulating or immunosuppressive medications, the type of vaccine received, and the time interval between their last vaccination and blood sampling. The types of vaccines were categorized into two groups according to their mechanisms, as follows: mRNA vaccines (BNT162b2 and mRNA-1273) and viral vector vaccines (ChAdOx1-S/nCoV-19 and Ad26.COV2.S).

This study was approved by the institutional review board of Samsung Medical Center (IRB number: 2021-09-118), and all participants provided written informed consent.

### 2.2. Antibody Detecting

To evaluate the anti-SARS-CoV-2-specific immunoglobulin G (IgG) from patients, each patient’s serum was immediately separated from the SST (serum separation tube, BD, NJ, USA) within two hours after blood collection, and the aliquot was kept in −80 °C until use. The anti-SARS-CoV-2 QuantiVac enzyme-linked immunosorbent assay (ELISA) kit (Euroimmun, Lübeck, Germany) was used with a 1:101 dilution of each patients’ serum, which was duplicated. The kit contained a plate coated with the recombinant S1 domain of the spike protein of SARS-CoV-2 and serial concentrations of the first WHO international standard for anti-SARS-CoV-2 immunoglobulin (NIBSC code 20/136) as calibrators. The standard curve, from which the concentration of antibodies in the samples can be determined, is obtained using point-to-point plotting of the extinction readings measured for 6 calibration sera against the corresponding units (linear/linear). Following the incubation of patients’ sera and enzyme conjugates, the absorbance was detected using a developed substrate and was measured using an xMark Microplate spectrophotometer (Bio-rad Inc., Hercules, CA, USA) at 450 nm. Following the manufacturer’s instructions, the antibody test results are presented in relative unit/mL (RU/mL), with interpretations as follows: <8 RU/mL was negative, ≥8 to <11 RU/mL was borderline, and ≥11 RU/mL was positive. The result from this ELISA kit confirmed 100% correlation with PRNT50 (plaque reduction neutralization test) and serial dilutions of the first WHO International Standard for anti-SARS-CoV-2 immunoglobulin (NIBSC code: 20/136), with an R2 value of 0.99 as reported by the manufacturer. The sensitivity was 90.3% 10 days after infection and 93.2% 21 days after infection, whereas the specificity was 99.8% [29].

### 2.3. Statistical Analysis

Baseline characteristics were compared between the healthy controls and the patient group, and appropriate summary statistics were incorporated. The categorical variables are shown as frequencies and percentages, and the continuous variables are shown as means and standard deviations (SDs) or medians and interquartile range (IQR) values, according to the normal distribution. The differences between the categorical variables were analyzed using Chi-square tests or Fisher’s exact tests. The differences between the continuous variables were analyzed using parametric student’s *t*-tests and non-parametric Mann–Whitney U tests. The correlation between test latency and antibody titer was calculated using Spearman’s correlation coefficient and is depicted in a scattered plot. The comparison of antibody titers, according to the type of vaccines and immunosuppressive or immunomodulating drugs, was conducted using Kruskal–Wallis tests and is depicted using box plots. Multiple linear regression analysis was used to examine the effect of various factors on antibody titer, including age, the time interval between the last vaccine dose and blood sampling, the use of immunomodulating/immunosuppressive agents, the administration of the mRNA vaccine, and having booster vaccines after standard doses. All tests were two-tailed, with *p* values of ≤0.05 considered significant. All statistical analyses were performed using SPSS Statistics 29.0. or R software version 4.2.1 (R Foundation for Statistical Computing, Vienna, Austria).

## 3. Results

### 3.1. Baseline Characteristics of Patients with Neuroimmunological Disorders and Healthy Participants

A total of 81 subjects were enrolled, including 25 patients and 56 healthy participants. Of the patients with neuroimmunological disorders, MS was the most common diagnosis (ten patients), followed by MG (seven patients). Six patients with NMOSD, one patient with optic neuritis (ON), and one patient with polymyositis (PM) were also included in this study. The patient group had a significantly higher female proportion (84.0% vs. 58.9%, *p* = 0.027), and the mean age at sampling of the patient group was greater than that of the control group (47.0 ± 12.1 years vs. 38.7 ± 11.7 years, *p* = 0.004). The time interval between vaccination and blood sampling was shorter in the patient group (84.0 [IQR 64.5–99.5] days vs. 94.0 [IQR 89.0–164.0] days, *p* = 0.037). There was a significant difference in the types of vaccines used between the patient group and the healthy control group, with a higher proportion of mRNA vaccines administered in the patient group (72.0% vs. 33.9%, *p* = 0.004). Only eight subjects received booster shots at the time of sampling (three patients and five healthy participants). The mean anti-SARS-CoV-2 antibody titer in the patient group was lower than that of the healthy controls (58.4 ± 49.2 RU/mL vs. 81.7 ± 47.3 RU/mL, *p* = 0.045). A comparison of the baseline characteristics and antibody results between the patient group and the healthy controls is shown in Table 1.

### 3.2. The Factors Influencing the Levels of SARS-CoV-2 Antibody Titers

**Figure 1:**
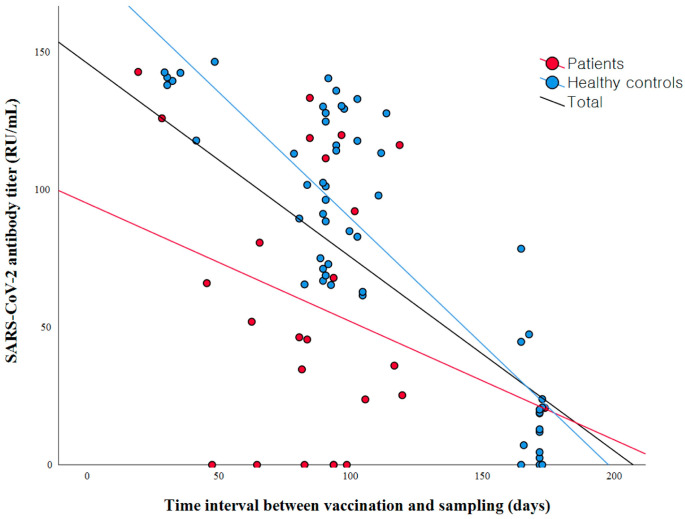
Scatterplot depicting the correlation between SARS-CoV-2 antibody titers and the time intervals between vaccination and sampling. A robust negative correlation was observed in the control group (red line, r =−0.713, *p* < 0.001), whereas no substantial correlation was observed in the patient group (blue line, r = −0.189, *p* = 0.365) between the time interval from vaccine administration to blood sampling and antibody titers.

Longer time intervals between vaccination and blood sampling were associated with lower concentrations of COVID-19 antibodies (r = −0.606, *p* < 0.001) (Figure 1).

**Figure 2:**
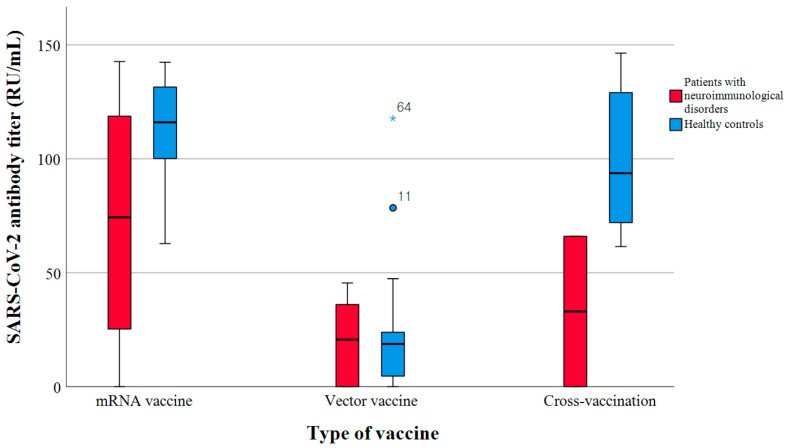
Comparison of SARS-CoV-2 antibody titers between patients with neuroimmunological disorders who were administered immunomodulating agents (red box) and healthy volunteers (blue box). The antibody titers were higher in individuals who received mRNA vaccines.

The control group exhibited a robust negative correlation, whereas no substantial correlation was observed in the patient group between the time interval from vaccine administration to blood sampling and antibody titers (r = −0.713, *p* < 0.001 vs. r = −0.189, *p* = 0.365). The antibody titers were different according to the types of vaccines administered (Figure 2).

**Figure 3:**
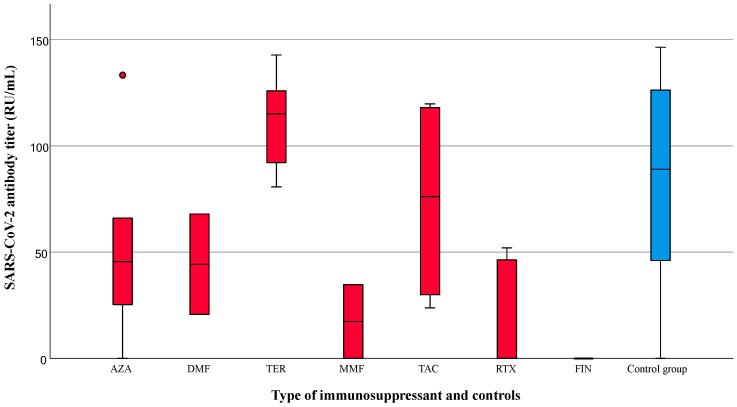
SARS-CoV-2 antibody titers according to the type of immunosuppressant administered. The median antibody titers (IQR) for patients (red box) using AZA, DMF, TER, MMF, TAC, RTX, and FIN and for healthy controls (blue box) were 45.5 (25.3–66.0), 44.3 (20.7–67.9), 115.0 (92.1–125.9), 17.3 (0.0–34.7), 76.1 (29.9–118.0), 0.0 (0.0–46.3), 0.0 (0.0–0.0), and 89.0 (46.0–126.2), respectively. AZA, azathioprine; DMF, dimethyl fumarate; TER, teriflunomide; MMF, mycophenolate mofetil; TAC, tacrolimus; RTX, rituximab; FIN, fingolimod.

Considering all participants, the SARS-CoV-2 antibody titers were significantly different according to the type of vaccination (mRNA vaccine, 113.0 [IQR 64.2–128.5]; vector vaccine, 18.9 [IQR 21.9–38.2]; cross-vaccine 90.3 [IQR 68.3–128.4], *p* < 0.001). Within the patient group, the individuals who received only mRNA vaccines exhibited the highest SARS-CoV-2 antibody titers, whereas those who received vector vaccines had the lowest titers (mRNA vaccine, 74.3 [IQR 25.3–118.7] RU/mL; vector vaccine, 20.7 [IQR 0.0–36.0] RU/mL; cross-vaccine, 33.0 [IQR 0.0–65.7] RU/mL; *p* = 0.092). In the control group, the patients who received vector vaccines displayed the lowest antibody titer compared to the mRNA or cross-vaccinated group (mRNA vaccine, 116.0 [IQR 100.1–131.5] RU/mL; vector vaccine, 18.7 [IQR 4.6–23.9] RU/mL; cross-vaccine, 74.3 [IQR 25.3–118.7] RU/mL, *p* < 0.001). The antibody titers based on the type of immunomodulatory drugs are displayed in Figure 3.

Patients taking mycophenolate mofetil, rituximab, or fingolimod exhibited lower concentrations of anti-SARS-CoV-2 antibody (17.3 [IQR: 0.0–34.7], 0.0 [IQR: 0.0–46.3], and 0.0 [IQR: 0.0–0.0] RU/mL, respectively). Multivariate testing for the independent factors that influenced the antibody titers showed that the time interval between last vaccination and sampling (B = −0.723, 95% CI: −0.926–−0.520, *p* < 0.001), the use of immunomodulating therapy (B = −49.503, 95% CI: −66.663–−32.344, *p* < 0.001), and the use of mRNA vaccines (B = 31.507, 95% CI: 14.602–48.411, *p* < 0.001) were independently associated with the antibody titers (Table 2).

## 4. Discussion

In this study, we conducted a prospective analysis of real-world data to investigate the titers of SARS-CoV-2 antibodies and their correlation with a range of clinical factors in patients with neuroimmunological disorders who are currently under immunomodulatory therapy. The SARS-CoV-2 antibody titers in the patients were observed to be lower compared to those in the healthy participants. In the multivariate regression analysis, the use of immunomodulating therapy was negatively associated with the SARS-CoV-2 antibody titer, whereas mRNA vaccine use was positively associated with the SARS-CoV-2 antibody titer.

Vaccine responses could be influenced under specific conditions by various treatments. Individuals receiving corticosteroids, immunosuppressive drugs, or anti-CD20 agents may exhibit a diminished response to COVID-19 vaccination [30]. Therefore, the efficacy of COVID-19 vaccination becomes a crucial issue in patients with neuroimmunological disorders undergoing these immunomodulating treatments. Our study showed the quantitative differences in vaccine response among patients with various neuroimmunological disorders using immunomodulatory agents. Given that SARS-CoV-2 antibody production was negatively correlated with the time interval between the last vaccine and blood sampling, our patient group showed lower antibody titers even with shorter time intervals. These findings suggest that the use of immunomodulating agents distinctly influences the efficacy of the SARS-CoV-2 vaccine. Notably, lower antibody titers were observed in patients receiving rituximab, mycophenolate mofetil, and fingolimod, which is consistent with previous studies [23,24,25,26]. König et al. reported that reduced humoral immunity was present in 82% and 80% of patients with MS treated with fingolimod and rituximab, respectively [31]. Mycophenolate mofetil, especially when combined with corticosteroids, could reduce the humoral response following COVID-19 vaccine administration. However, the administration of a third vaccine dose has shown improved seroconversion rates [26].

CD20-depleting therapies, although extensively utilized in the management of neuroimmunological disorders, are associated with an increased risk of severe COVID-19 infection [32] and a dampened humoral response to COVID-19 vaccination [23,31,33]. The development of protective neutralizing antibodies and the response to vaccination are expected to be attenuated until naive B-cells repopulate [34]. In our study, 20% of patients (five out of twenty-five) were undergoing rituximab treatment, all of whom were diagnosed with NMOSD. These patients exhibited diminished SARS-CoV-2 antibody titers. Three out of five patients showed a complete absence of antibody production. Additionally, even after booster vaccinations were administered, one patient still exhibited no antibody response (Table 3).

These findings suggest that in some patients, despite additional vaccination, antibody production may remain insufficient. This highlights the potential need for individual adjustment of the COVID-19 vaccination schedule, especially for those undergoing anti-CD20 therapy. In regards to the type of vaccine, both the patient and control groups demonstrated a consistent trend of lower titers in the vector vaccine group compared to those who received mRNA vaccines only or those in the cross-vaccinated group. This correlates with previous reports showing the relatively higher efficacy of mRNA vaccines versus viral vector vaccines [35]. The current guidelines for COVID-19 vaccination in South Korea do not allow cross-vaccination [36]. However, considering the higher titers exhibited in subgroups that received at least one dose of an mRNA vaccine (mRNA group and cross-vaccinated group) compared to those who received only vector vaccines, it may be more effective for immunocompromised individuals to receive an mRNA vaccine even if they previously received a vector vaccine.

There is a complex interplay between infection, vaccination, and immunomodulating therapies. The management of these interactions is critical in reducing infection risks and optimizing vaccination benefits [37]. Our study reinforces and expands the prevailing understanding of the relationship between immunomodulating therapies and COVID-19 vaccination and underscores a crucial consideration for clinicians when informing patients about the potential risk of reduced humoral immunity following vaccination.

Our study has several limitations. Firstly, we evaluated patients with various neurological disorders who were using immunomodulatory drugs, resulting in a relatively small number of patients in each specific disorder group. Previous studies have been conducted on a single disease, such as multiple sclerosis or myasthenia gravis [38]. However, as we compared the antibody titers among different medications, we believe that our results provide meaningful insights that can be applied to patients with a diverse range of neuroimmunological disorders using immunomodulatory drugs in real-world outpatient clinic settings. Second, the time interval between vaccination and sampling was not strictly controlled, and we did not investigate serial changes in the antibody titers. The time interval between vaccination and blood sampling is a critical determinant in evaluating antibody titers. Our study also showed that this time interval was independently associated with the titers. Therefore, to comprehensively understand the changes in antibody titers, serial antibody testing with controlled time intervals is necessary. Third, we did not check COVID-19 infection as an outcome of COVID-19 vaccination. The most important outcome of vaccination is infection and the severity of infection, not the antibody titers. Medalon et al. suggested that in patients with autoimmune disorders on immunosuppressive medications, T-cell responses might be preserved despite low antibody titers, which could potentially influence the severity of COVID-19 infection [39]. Further large follow-up studies are warranted. Finally, all the patients in our study completed the primary vaccine series; however, only a small number of patients received booster vaccinations. Recently, mRNA-based bivalent vaccines have been introduced and are currently recommended for immunocompromised patients as additional doses in Korea [36]. However, our study was conducted during a period when booster shots were not being strongly considered. Although we only had four cases of follow-up antibody tests after booster shots of the vaccine, we observed the formation of antibodies in two out of three individuals who initially tested negative after receiving the primary complete dose of the vaccine, except for one participant with NMOSD who received rituximab treatment. Therefore, individualized vaccination plans and close monitoring is needed for patients on specific immunomodulating medications for neuroimmunological disorders.

## 5. Conclusions

The results of this study indicate that the use of immunomodulating drugs could alter the efficacy of SARS-CoV-2 vaccines. Hence, physicians should consider this when managing patients with neuroimmunological disorders who are undergoing treatment with immunomodulating medications.

## Figures and Tables

**Table 1:** Baseline characteristics were compared between the healthy controls and the patient group.

	Total (*N* = 81)	pwNID (*N* = 25)	Healthy Controls (*N* = 56)	*p* Value
Female, *N* (%)	54 (66.7)	21 (84.0)	33 (58.9)	0.027
Age at sampling, year (mean ± SD)	41.3 ± 12.4	47.0 ± 12.1	38.7 ± 11.7	0.004
The time interval between vaccination and sampling, days (IQR)	93.0 (82.5–114.5)	84.0 (64.5–99.5)	94.0 (89.0–164.0)	0.037
Type of vaccination, *N* (%)				0.004
mRNA vaccine ^†^	37 (45.7)	18 (72.0)	19 (33.9)	
Vector vaccine ^‡^	22 (27.2)	5 (20.0)	17 (30.4)	
Cross-vaccination	22 (27.2)	2 (8.0)	20 (35.7)	
Clinical diagnosis, *N* (%)				
Myasthenia gravis		7 (28.0)		
Multiple sclerosis		10 (40.0)		
NMOSD		6 (24.0)		
Optic neuritis		1 (4.0)		
Polymyositis		1 (4.0)		
Medication, *N* (%)				
Azathioprine		5 (20.0)		
Dimethyl fumarate		2 (8.0)		
Teriflunomide		6 (24.0)		
Mycophenolate mofetil		2 (8.0)		
Tacrolimus		4 (16.0)		
Rituximab		5 (20.0)		
Fingolimod		1 (4.0)		
COVID-19 antibody titer, RU/mL	74.5 ± 48.8	58.4 ± 49.2	81.8 ± 47.3	0.045
Antibody positivity, *N* (%)	69 (85.2)	19 (76.0)	50 (89.3)	0.120

^†^ BNT162b2 and mRNA-1273 vaccines. ^‡^ ChAdOx1-S/nCoV-19 and Ad26.COV2.S vaccines. pwNID, patients with neuroimmunological disorders; NMOSD, neuromyelitis optica spectrum disorder.

**Table 2:** Multiple linear regression analysis of the factors affecting SARS-CoV-2 antibody titers.

	Univariate Test			Multivariate Test		
	B	95% CI	*p* Value	B	95% CI	*p* Value
Age, per 1 year increase	−0.311	−1.193~0.571	0.485	−0.356	−0.974~0.261	0.254
Time interval between last vaccination and blood sampling, per 1 day increase	−0.706	−0.913~−0.498	<0.001	−0.723	−0.926~−0.520	<0.001
Use of immunomodulating agents	−23.410	−46.333~−0.488	0.045	−49.503	−66.663~−32.344	<0.001
Use of mRNA vaccines only	33.896	13.455~54.338	0.001	31.507	14.602~48.411	<0.001
Having booster vaccination	26.882	−9.024~62.789	0.140	9.547	−17.654~36.748	0.487

**Table 3:** Clinical characteristics of the patients on rituximab therapy.

No.	Age	Sex	Diagnosis	Days after Last RTX Injection	Cumulative Dose of RTX (mg)	Vaccine			Days after Last Vaccination	SARS-CoV-2 IgG Titer (RU/mL)
						1st	2nd	3rd		
1	41	Female	NMOSD	56	2400	BNT162b2	BNT162b2		62	51.979
2	46	Female	NMOSD	63	3000	BNT162b2	BNT162b2		93	0
3	60	Female	NMOSD	84	5600	ChAdOx1	ChAdOx1		98	0
4	41	Female	NMOSD	152	2400	BNT162b2	BNT162b2	BNT162b2	80	46.326
5	60	Female	NMOSD	173	5600	ChAdOx1	ChAdOx1	BNT162b2	82	0

NMOSD, Neuromyelitis optica spectrum disorder; RTX, Rituximab.

## Data Availability

The data that support the findings of this study are available from the corresponding author upon reasonable request.
